# Oxytocin activity is not linked to out-group prosociality in wild bonobos

**DOI:** 10.1038/s41598-025-00209-w

**Published:** 2025-06-03

**Authors:** Leveda Cheng, Liran Samuni, Tobias Deschner, Martin Surbeck

**Affiliations:** 1 Present Address: Department of Human Evolutionary Biology, Harvard University, Cambridge, MA USA; 2 Present Address: Department of Evolutionary Anthropology, Duke University, Durham, NC USA; 3 Present Address: Max Planck Institute for Evolutionary Anthropology, Leipzig, Germany; 4 Cooperative Evolution Lab, German Primate Center, Göttingen, Germany; 5 Comparative BioCognition, Institute of Cognitive Science, University of Osnabrück, Osnabrück, Germany; 6 Department of Human Behavior, Ecology and Culture, Max Planck Institute for Evolutionary Anthropology, Leipzig, Germany

**Keywords:** Neuropeptide, *Pan paniscus*, Intergroup tolerance, Prosociality, Collective defense, In-group, Out-group, Animal behaviour, Behavioural ecology, Animal physiology, Endocrinology

## Abstract

In many group-living species, cooperative group defense is crucial to the reproduction and survival of group members. In humans and chimpanzees, this adaptive behavior is regulated by oxytocin, a highly conserved neurohormone. In humans, oxytocin can also enhance prosocial attitudes towards out-group individuals and reduces xenophobia. While the role of oxytocin in supporting cooperative group defense is likely evolutionarily ancient, it is unclear to what extent oxytocin’s role in promoting out-group prosociality is conserved. Bonobos, our closest living relatives together with chimpanzees, can provide valuable insights into this question, because they are not known to engage in collective group defense but instead exhibit tolerance and prosocial behaviors across groups. Through examining variation in bonobo cooperative behavior, specifically coalition formation, we reinforce the idea that bonobo coalitions do not serve as a form of group defense. Despite increased competition, bonobos formed fewer coalitions in the presence of out-groups. Further, bonobo coalitions included both in- and out-group partners, reflecting reduced xenophobia and between-group cooperation. Physiologically, neither females nor males showed increased oxytocin activity with out-group presence. This suggests that, unlike in humans, oxytocin is not involved in regulating out-group prosociality in bonobos.

## Introduction

From cells to societies, cooperation is widespread across biological systems. How natural selection shapes diverse forms of cooperation across taxa remains a central question in biology^1^. In humans and other social animals, costly cooperation with uncertain returns is evident in the context of between-group conflicts. Cooperative group defense is key to success, survival, and reproduction of individuals within a group^2–7^. Consequently, between-group conflict has been identified as a driver of within-group cooperation in humans^8,9^. Across nonhuman taxa, between-group conflict can have immediate and lasting effects on in-group affiliation and cooperation, such as in birds, fish, and mammals^10,11^. At the individual level, participation in between-group conflicts can carry substantial costs, including increased stress^12,13^, and risks of injury and death^14^. Decisions to cooperate with group members in this context depend on individual payoffs associated with between-group conflict^15–17^ and the strength of social relationships within the group^18^. At the group level, the degree of previous out-group threat can also influence within-group cooperation^19^. Most studies investigating the effect of between-group conflict on within-group cooperation have focused on species in which out-groups are considered a strong threat to the fitness of in-group members^14^. This limits our understanding of whether and how different degrees of out-group threat can generate different selection pressures on within-group sociality and cooperation.

One of the key physiological mechanisms underlying within-group social relationships and cooperation is oxytocin^20–22^. Oxytocin is a nonapeptide hormone produced in the hypothalamus and released into the bloodstream by the posterior pituitary. Thus, oxytocin levels can be measured in peripheral fluids like blood plasma, saliva, and urine^23^. The physiological and behavioral effects of oxytocin are evolutionarily conserved across mammals^22^. While the primary function of oxytocin is to regulate maternal behavior, birth, and lactation (e.g., humans, rodents, sheep^24^), it has been shown to facilitate bonding between unrelated individuals (e.g., human and nonhuman primates, voles, dogs:^22,25,26^). Furthermore, oxytocin is also known to support group cooperation through increasing in-group concern and cohesion, and reducing the likelihood of defection (e.g., humans and chimpanzees:^27–29^). However, the precise role of oxytocin in regulating in-group out-group interactions is debated^30,31^. Experimental studies on humans revealed that oxytocin elicits out-group hostility and defensive aggression only when out-group threat is eminent^27,32^. In contrast, natural observations on chimpanzees showed that oxytocin activity is linked to collective group defense, regardless of the level of potential imminent out-group threat^28^. In non-competitive contexts, oxytocin can promote human prosocial behaviors and cooperation across groups^33,34^. Taken together, the role of oxytocin in regulating cooperation during between-group interactions appears to be tied to the severity of between-group conflict and the benefits of within-group cooperation during between-group conflict.

To decipher the evolutionary preserved functions of the oxytocinergic system, we need to examine oxytocin activity when interactions with out-groups can be aggressive and prosocial. Bonobos (*Pan paniscus*), our closest living relatives alongside chimpanzees, may serve as comparative models for understanding oxytocin’s adaptive functions in intergroup contexts. Chimpanzees have predominantly hostile intergroup relationships and group members, particularly males, collectively defend group territory through border patrols and intergroup aggression. Like human intergroup violence, chimpanzee collective group defense can escalate into lethal intergroup aggression^7^. In both humans and chimpanzees, the oxytocinergic system serves as a biological mechanism supporting cooperative defense^27,28^. Bonobos, on the other hand, are similar to humans in terms of their capacity to act prosocially and cooperate with out-groups^35^. Interactions between bonobo groups are characterized by diverse social behaviors including socio-sexual behaviors, aggression, and prosocial behaviors, including grooming, and food sharing^36–41^. There are apparent sex differences in bonobo social strategies towards out-groups, with males being more aggressive than females^36^ and females being more involved in coalition formation and food sharing^40^ during intergroup encounters. Unlike chimpanzees, bonobos are not known to collectively defend group range. While bonobo groups maintain exclusive ranging areas, there can be extensive overlaps in the use of the habitat between groups^42^. Although there seems to be variation in the occurrence and severity of aggression during intergroup encounters within and across bonobo populations^39,43–45^, coalitionary killings have never been observed in any of the long-term study sites^46^. The juxtaposition of intergroup relationships between the two *Pan* species highlights bonobos’ out-group tolerance and reduced xenophobia, making bonobos a valuable model species to examine whether oxytocin’s function in promoting cooperation across groups, as seen in humans, is also conserved in bonobos.

According to the ‘tend-and-defend’ hypothesis^29^, oxytocin involvement in cooperative defense is evolutionary conserved in species in which out-groups pose a fitness-related threat that can be mitigated by cooperative defense. Given mild severity of intergroup aggression and apparently low levels of out-group threat experienced by bonobos, the benefits of cooperative defense and the significance of oxytocin in regulating such behavior are likely reduced relative to species with high-risk, potentially lethal between-group interactions. Following the ‘tend-and-defend’ hypothesis, we predict that the presence of an out-group will neither be associated with within-group cooperation nor oxytocinergic system activity in bonobos, unlike in humans or chimpanzees. Alternatively, the ‘out-group prosociality’ hypothesis states that oxytocin serves as a social reward signal motivating prosocial behaviors and cooperation between groups. Indeed, oxytocin facilitates prosocial attitudes across groups in humans^33,34^ and in bonobos, it may promote dyadic affiliative interactions with in-group members such as socio-sexual interactions^47^ and grooming^48^. Based on the ‘out-group prosociality’ hypothesis, we predict that the presence of an out-group will be associated with cooperation both within and between groups and higher oxytocin levels in bonobos. Given the prevalence of female-female coalitions and female active involvement in affiliative interactions during intergroup encounters^41^, we also predict that females may have a stronger response in oxytocin activity during intergroup interactions compared to males.

To investigate the role of oxytocin in largely tolerant intergroup encounters in bonobos, we use behavioral observations and urine samples from 15 males and 23 females from two neighboring groups at the Kokolopori Bonobo Reserve, Democratic Republic of Congo. The two study groups meet frequently^42^ and these encounters are often tolerant and not transient^49^. Additionally, the two groups regularly engage in prosocial exchanges including grooming and food-sharing^40^. Here, we first characterized bonobo coalition formation (in which two or more individuals jointly attack the same individual at the same time) during intergroup encounters in greater detail, examining whether bonobos, like humans and chimpanzees, participate in collective group defense. Then, we investigated the effect of the presence of out-group individuals on variation in individual within-group cooperation tendencies and urinary oxytocin levels while comparing both sexes. We operationalized within-group cooperation tendency as the likelihood of individuals to participate in coalitionary aggression with in-group partners, considering all individuals that were present at the time of the aggression. We measured oxytocin concentration in urine samples using a method that has been previously validated in bonobos^47^.

## Results

### Within-group and between-group coalitions

We observed 347 coalitionary aggressions between members of the same group (out of 35,253.5 h of observations). Of these coalitionary aggressions, 206 occurred in the absence of out-group individuals (out of 29,687.5 h of observations) and 141 during out-group presence (out of 5566 h of observations). During out-group presence, the majority of coalitionary aggressions with in-group partners were directed at out-group (N = 108, 76.6%) rather than in-group individuals (N = 33, 23.4%; Table 1). Further inspection into the actor and receiver of coalitionary aggressions during out-group presence revealed that these were mostly female-female coalitions (N = 124, 87.9%) and males were the main target (N = 91, 64.5%). Moreover, males never formed coalitions with other males to jointly attack females.

**Table 1:** Comparison of the number of within-group coalitions during out-group absence and out-group presence.

Type of coalition	Target group	Target sex	Out-group absent		Out-group present	
			N	%	N	%
Female-female	In-group	Female	11	5.3	6	4.3
	In-group	Male	83	40.3	22	15.6
	Out-group	Female	-	-	43	30.5
	Out-group	Male	-	-	53	37.6
Male-male	In-group	Female	2	1.0	0	0
	In-group	Male	39	18.9	0	0
	Out-group	Female	-	-	0	0
	Out-group	Male	-	-	4	2.8
Mixed-sex	In-group	Female	2	1.0	0	0
	In-group	Male	71	34.5	5	3.5
	Out-group	Female	-	-	1	0.7
	Out-group	Male	-	-	7	5.0

In addition to within-group cooperation, we observed cooperation between the two study groups in 63 coalitionary aggressions (51 female-female coalitions and 12 mixed-sex coalitions). Like coalitionary aggressions with in-group partners, the main target of these coalitionary aggressions with out-group partners were males (N = 49, 77.8%).

### Within-group coalition participation rates and size

We characterized bonobo coalitions by examining average rates of coalition participation for females and males as well as the average number of coalition partners. We included all opportunities for in-group individuals to participate in coalitionary aggressions by considering all in-group individuals present at the time of a coalitionary aggression as potential participants and marking whether they joined the coalition. Therefore, the same coalitionary aggression event was represented multiple times in the dataset, one for each potential participant. Considering all in-group individuals present in the party as potential partners, a female on average participated in 19.3% of the coalitionary aggressions when the out-group was absent, and 18.5% when the outgroup was present. In contrast, a male on average participated in 22.8% of the coalitionary aggressions when the out-group was absent, compared to only 4% when the outgroup was present. Furthermore, the size of bonobo coalitions was small both when the out-group was absent and present (out-group absent: mean ± SD = 2.4 ± 0.7; out-group present: mean ± SD = 2.4 ± 0.7). Comparing the number of coalition partners a given male/female has excluding that male/female, females had more partners (out-group absent: mean ± SD = 1.7 ± 0.9; out-group present: mean ± SD = 1.6 ± 0.8) than males (out-group absent: mean ± SD = 1.3 ± 0.6; out-group present: mean ± SD = 1.3 ± 0.7). Overall, bonobo coalitionary aggressions during intergroup encounters is very different from chimpanzee collective group defense in terms of its nature and group-level participation. Chimpanzee collective group defense via border patrols and intergroup encounters, where individual participation rates are considerably high (around 50% for females in Taï and 85% for males in Taï and Ngogo) and all border patrol participants jointly attack the out-group during intergroup encounters (on average 8 to 13 participants in Taï and Ngogo, respectively)^18,50^.

### Individual likelihood to participate in coalitionary aggressions with in-group partners

**Fig. 1:**
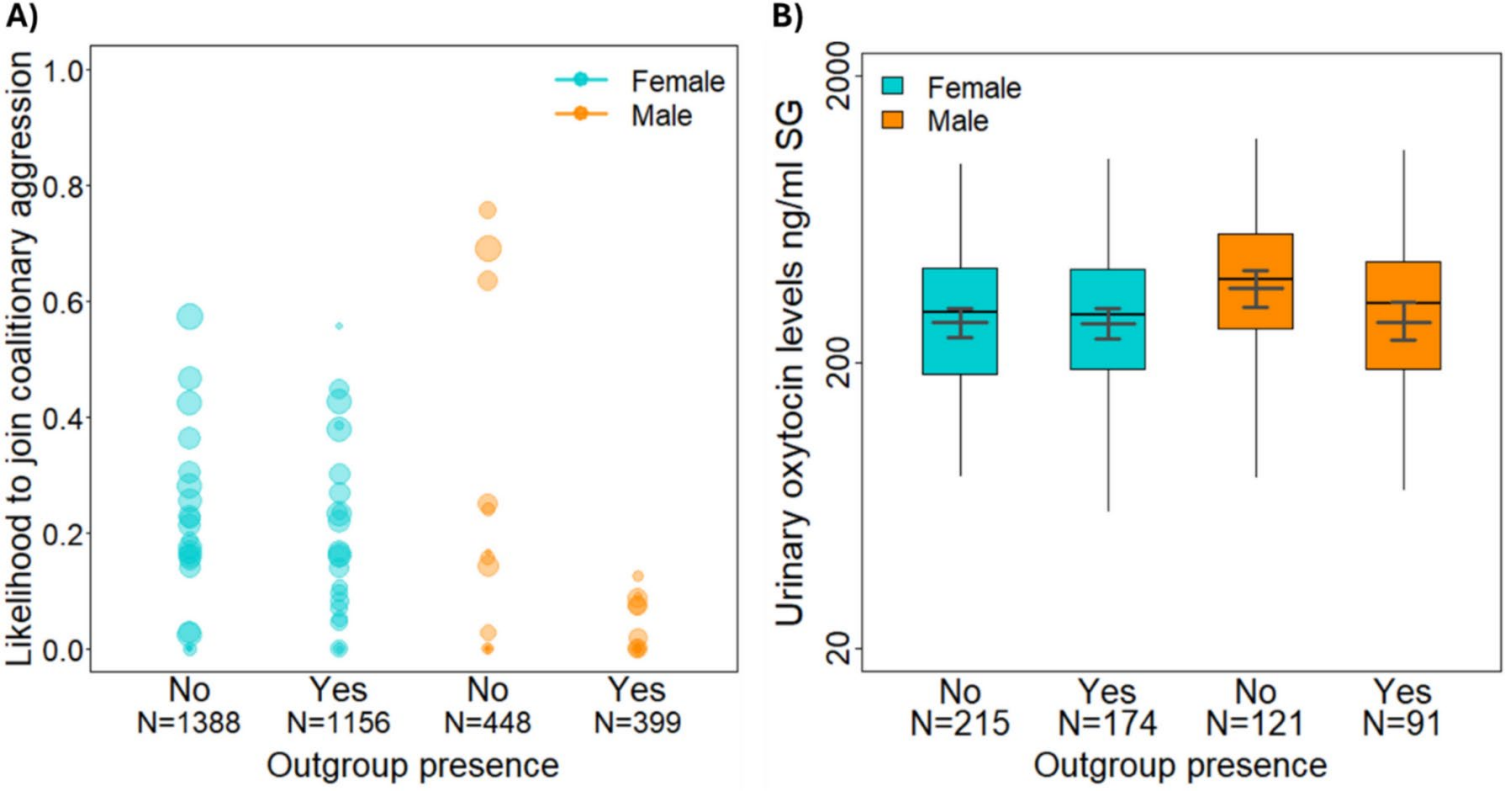
(**A**) Likelihood to join coalitionary aggressions with in-group partners in relation to out-group presence (312 coalitionary aggressions, 38 individuals, 25 recipients, 420 dyads; total N = 3391). Circles represent the average likelihood of an individual to join coalitionary aggressions with in-group partners. The size of the circles indicates the number of observations per individual. (**B**) Female and male urinary oxytocin levels in relation to out-group presence (601 samples, 33 individuals, 251 days). Shown are medians (thin horizontal lines), quartiles (boxes), percentiles (2.5 and 97.5%; vertical lines), as well as the fitted model (thick horizontal lines) and its 95% confidence intervals (error bars).

We examined whether the likelihood for an individual to participate in coalitionary aggressions with in-group partners was associated with the presence of out-group individuals and whether this association varied between the sexes. During out-group presence, coalitionary aggressions with in-group partners targeted both in- and out-group individuals. Since we wanted to examine within-group cooperation in the context of cooperative group defense, during out-group presence we only considered coalitionary aggressions that targeted out-group individuals, whereas during out-group absence we included all coalitionary aggressions. Our final dataset comprised 3391 number of coalition participation opportunities in which 312 coalitionary aggressions occurred among 38 potential participants and 420 dyads. The full model comprising the interaction of out-group presence and individual sex, as well as additional control predictors (see Methods) explained significantly more variance than the null model, which was identical to the full model but lacked the control predictors (generalized linear mixed model (GLMM) likelihood ratio test: χ^2^ = 19.951, *df* = 2, *p* < 0.001; Fig. 1A and Table 2). The interaction between out-group presence and individual sex was significant (GLMM: estimate ± SE = -2.579 ± 0.829, *p* = 0.003). When comparing patterns of coalitionary aggression participation during out-group absence and presence, we found that both sexes were less likely to participate in coalitionary aggressions with in-group partners when out-group individuals were present (post hoc analysis: female: *z*-value = -3.224; *p* = 0.006; male: *z*-value = -4.593; *p* < 0.001). When comparing patterns of participation between the sexes, we found that females were more likely to participate in coalitionary aggressions than males when out-group individuals were present (*z*-value = -3.951; *p* < 0.001), whereas the likelihood to participate in coalitionary aggressions were comparable between the sexes when the out-group was absent (*z*-value = -0.997; *p* = 0.734).

**Table 2:** GLMM examining the effect of out-group presence and individual sex on likelihood to participate in coalitionary aggression.

Term	Coded level	Est	SE	CI _lower_	CI _upper_	Chisq	*p*
Intercept	-	–3.976	0.474	–4.585	–3.518	-	-
Test predictors:							
Out-group presence x Sex		–2.579	0.829	–3.086	–1.028	8.694	0.003
(No out-group presence, female)	Out-group presence, female	–1.103	0.342	–1.458	–0.645	-	-
(No out-group presence, female)	No out-group presence, male	–0.807	0.808	–1.926	–0.355	-	-
(Out-group presence, male)	No out-group presence, male	3.662	0.797	2.593	4.506	-	-
(Out-group presence, male)	Out-group presence, female	3.365	0.852	2.720	4.922	-	-
Control predictors:							
Individual rank^a^ x Recipient rank^b^		–0.018	0.232	–0.208	0.571	3.647	0.056
Individual rank^a^	-	0.776	0.298	0.160	1.050	-	-
Recipient rank^b^	-	0.043	0.206	-0.366	0.271	-	-
Recipient sex (female)	male	0.617	0.433	0.377	1.361	5.417	0.020

Reference levels are shown in parenthesis.

^a^z-transformed, original mean ± SD = 0.65 ± 0.3 (range from 0 to 1, with 1 being the highest ranking).

^b^z-transformed, original mean ± SD = 0.45 ± 0.33 (range from 0 to 1, with 1 being the highest ranking).

### Variation in urinary oxytocin

We examined the effect of the presence of out-group (within the time window for oxytocin secretion), individual sex, and their interaction on urinary oxytocin levels. The full model comprising the interaction of out-group presence and individual sex, as well as other control predictors (see Methods) was significantly different from the null model, which was identical to the full model but lacked the control predictors (linear mixed model (LMM) likelihood ratio test: χ^2^ = 10.430, *df* = 3, *p* = 0.015; Fig. 1B and Table 3). The interaction of out-group presence and individual sex was significant (LMM: estimate ± SE = -0.256 ± 0.109; *p* = 0.031). Inconsistent with predictions for the ‘out-group prosociality’ hypothesis, oxytocin levels did not increase with out-group presence in either sex. Rather, males had lower oxytocin levels in the presence of the out-group (*p* = 0.010) whereas females had similar oxytocin levels regardless of out-group presence (*p* = 0.859). When comparing oxytocin patterns between the sexes, males had higher oxytocin levels than females when the out-group was absent (*p* = 0.006), but male and female oxytocin levels were comparable when the out-group was present (*p* = 0.839). We additionally accounted for the effect of grooming on individual oxytocin activity (i.e., whether the sampled individual was grooming or groomed by others within 15 and 60 min of sample collection) and did not find a significant effect of grooming (*p* = 0.725).

**Table 3:** LMM examining the effect of out-group presence and individual sex on urinary oxytocin levels, log-transformed.

Term	Coded level	Est	SE	CI _lower_	CI _upper_	*t*	*df*	*p*
Intercept	-	5.560	0.075	5.418	5.707	-	-	-
Test predictors:								
Out-group presence x Sex		–0.256	0.109	–0.473	–0.034	-2.250	22.813	0.031
(No out-group presence, female)	Out-group presence, female	–0.015	0.078	–0.163	0.137	-0.189	38.504	0.859
(No out-group presence, male)	Out-group presence, male	–0.271	0.097	–0.469	–0.081	-2.792	34.117	0.010
(No out-group presence, female)	No out-group presence, male	0.269	0.089	1.000	0.435	3.037	31.405	0.006
(out-group presence, female)	Out-group presence, male	0.013	0.092	–0.156	0.191	0.138	49.993	0.839
Control predictors:								
Grooming (no)	Yes	0.024	0.076	–0.121	0.180	0.311	551.217	0.725
Dominance rank^a^	-	0.011	0.035	–0.059	0.075	0.329	22.747	0.782
Number of in-group individuals^b^	-	–0.087	0.028	–0.143	–0.035	-3.074	582.063	0.002
Group (Ekalakala)	Kokoalongo	0.199	0.078	0.046	0.351	2.565	28.185	0.023
Urine amount								
(500 μl)	200 μl	–0.199	0.072	–0.341	–0.057	-2.774	10.507	0.022
(500 μl)	300 μl	–0.071	0.137	–0.346	0.205	-0.516	16.113	0.626
(500 μl)	400 μl	0.136	0.121	–0.103	0.391	1.125	7.519	0.381

Reference levels are shown in parenthesis.

^a^z-transformed, original mean ± SD = 0.59 ± 0.32 (range from 0 to 1, with 1 being the highest ranking).

^b^z-transformed, original mean ± SD = 7.96 ± 3.80.

## Discussion

We augment previous evidence that bonobo coalitionary aggression during intergroup encounters is not restricted to out-group defense as individuals cooperate with both in- and out-group individuals^40,41^. During intergroup encounters, coalitions with group members mostly targeted out-group individuals. However, these coalitionary aggressions are small in scale and individual participation rates are low. When compared to males, females have more coalition partners and higher rates of coalition participation when out-groups were present. Consistent with the idea that female bonobos actively foster tolerance between groups^38^, we find that females cooperate with the out-group by regularly forming agonistic coalitions with out-group individuals to attack males. On the mechanistic level, between-group interactions with mild conflict and high tolerance are not associated with increased oxytocin secretion in either sex. Therefore, our results support the ‘tend-and-defend’ hypothesis, which predicts that the presence of out-groups will not be associated with increased within-group cooperation nor oxytocinergic system activity in bonobos due to low out-group threat. We do not find support for the ‘out-group prosociality’ hypothesis, as oxytocin does not seem to regulate tolerance and cooperation between groups in bonobos. This indicates that bonobos do not ‘tend-and-defend’ and that oxytocin’s function in promoting out-group prosociality is lost in the bonobo lineage. To identify whether oxytocin’s out-group prosociality function is unique to humans, we need further studies on oxytocin reactivity in the context of out-group prosociality in other species.

Intergroup dynamics at Kokolopori are largely comparable to those in Wamba, a bonobo research site located 90 km away. In both sites, neighboring groups have considerably overlapping home ranges and interact frequently^51,52^. Furthermore, the frequency of intergroup encounters is closely tied to seasonal fluctuations in fruit availability in the two sites^44,45^. On the behavioral level, interactions between groups are largely tolerant, with individuals spending up to 30–35% of the observation days in association with neighboring groups^36,45^. Males are the more aggressive sex during intergroup encounters in Kokolopori^36^ and Wamba^41^, but Kokolopori males mostly contest individually rather than cooperatively^53^. Despite being more aggressive, males are often the target of coalitionary aggressions, especially by females. In both Kokolopori and Wamba, females even forge cooperative ties between groups to attack in-group males^38,41,52^. These between-group cooperative ties may confer benefits to all females, allowing them to out-compete males in intersexual conflicts and maintain high social status in a male-philopatric society. However, different from Wamba^41^, Kokolopori females regularly form coalitions to attack out-group individuals. Observations from other bonobo populations such as Lomako and LuiKotale reveal further variation in intergroup dynamics within bonobos, with intergroup encounters occurring less frequently at those sites and between-group interactions being less tolerant^39,54^.

Our findings that the presence of out-groups was not associated with higher oxytocin levels in bonobos contrast findings from humans^27^ and chimpanzees^28^. In chimpanzees, increases in endogenous oxytocin promotes participation in territorial border patrols and between-group conflict in males and females^28^. The difference in oxytocin patterns observed in bonobos and chimpanzees are unlikely due to methodological differences in sampling and analyses because both studies followed the same protocol to collect, store, and analyze urine samples, and urine sample analysis was conducted in the same endocrinology laboratory. Rather, this difference in findings is likely related to key differences in bonobo and chimpanzee intergroup relationships, especially the level of out-group threat and degree of group cooperation experienced by the two species. Chimpanzee between-group interactions are predominantly hostile and potentially deadly^46^. Our previous work demonstrates that competition occurs between bonobo groups and both sexes incurred physiological costs of competition (higher cortisol levels) during intergroup encounters^36^. Despite costs of competition, bonobos do not form more coalitions to defend against the out-group. Instead, they maintain largely tolerant relationships with neighboring groups^43,52^. Overall, decades of observations of the two species across populations indicate that the level of out-group threat is substantially lower in bonobos relative to chimpanzees^55^. The marked differences in bonobo and chimpanzee intergroup dynamics likely reflect differences in selection pressures in the two *Pan* species^56–58^.

Oxytocin acts as a biological mechanism underlying in-group out-group interactions in humans by promoting tolerance, trust, empathy, and generosity towards the in-group^59,60^, as well as protection of in-group members against out-group threat^32,61^. This ‘tend-and-defend’ function of oxytocin likely has deep evolutionary roots, especially in social species that experience strong threat from outsiders (where interactions with outsiders can be deadly)^29^. For example, oxytocin facilitates defensive aggression against intruders of a different strain in mice^62^, and elicits sentinel behavior, a costly behavior that protects own group against threat from predators and outgroups, in meerkats^63^.

There is ample evidence demonstrating that oxytocin plays a crucial role in maintaining social relationships across taxa^20^. For instance, oxytocin regulates social preference and reduces social avoidance in rats and mice^64^. In horses, oxytocin alters social preferences by increasing social proximity of previously rarely associated partners but reducing proximity of previously closely associated partners ^65^. In contrast, oxytocin promotes social proximity with familiar conspecifics in African lions, and reduces vigilance behavior towards outsiders^66^. Oxytocin also increases affiliative interactions and approaches towards familiar conspecifics in naked mole rats^67^ and domestic dogs^25^. Within primates, oxytocin modulates within-group affiliative behaviors like grooming and other behaviors that are key to strengthening social relationships in capuchins^68^, bonobos^47,48^, chimpanzees^28,69^, and humans^22,70^. Given that bonobos engage in various forms of affiliative social interactions with in- and out-group individuals during intergroup encounters^36–38,40,41^, it is unexpected that oxytocin activity does not increase during tolerant intergroup encounters. It may be that oxytocin only functions to promote prosociality in strictly non-competitive situations^33,34^. Alternatively, oxytocin is known to increase the salience of positive and negative social interactions through its interaction with the dopaminergic system^71^. It is possible that oxytocin reactivity is suppressed in the context of intergroup interactions among bonobos due to its maladaptive response to out-group defense, and this suppression has cascading effects on oxytocin expression in out-group prosociality.

Between-group conflict is considered a major selection pressure shaping the evolution of within-group cooperation, with the oxytocinergic system being a key underlying proximate mechanism of cooperative defense. However, the link between oxytocin and cooperation is context dependent. Here, we reinforce this notion by showing that oxytocin activity is not associated with between-group interactions that are largely tolerant and prosocial, but collective group defense is limited and lethal coalitionary intergroup aggression is absent. This implies that oxytocin may only be co-opted to support group cooperation during between-group conflict when such cooperation is vital to survival, and fitness of group members is interdependent.

## Methods

### Study population and site

All data were collected from two neighboring communities (Ekalakala and Kokoalongo) in the Kokolopori Bonobo Reserve, Democratic Republic of Congo. The two groups had distinct home ranges that substantially overlapped (exclusive area: Ekalakala: 17.9 km^2^; Kokoalongo: 26.46 km^2^: Overlap: 29.46 km^2^)^49^. We used long-term data collected between 2016 and 2021 for the coalitionary aggression dataset and a subset of data collected between 2016 and 2018 for the oxytocin dataset. Over the years, there were between 15 and 17 individuals in Ekalakala and between 35 and 45 individuals in Kokoalongo. We included data from individuals of 10 years and older in this study (female N = 18–23; male N = 7–16).

### Data collection

#### Agonistic interactions

Due to the fission–fusion dynamics of bonobo societies, group members were not always observed together. To maximize the chance of observing intergroup encounters, we continuously followed the largest subgroup (referred to as party) of bonobos throughout the day. We recorded all occurrences of dyadic and polyadic agonistic interactions that occurred in the party^72^, noting the identity of the aggressors and recipients.

#### Out-group presence

We determined out-group presence based on the presence of two or more out-group individuals in the followed party. This thus excludes instances of temporary visits from single out-group individuals.

#### Urinary sample collection and analysis

We collected urine samples opportunistically and non-invasively during daily party follows. All samples were shipped on dry ice to the Endocrinology Lab at the Max Planck Institute for Evolutionary Anthropology and stored at − 20 °C until extraction. We extracted urinary oxytocin with Sep-Pak cartridges, and then assayed using the commercially available enzyme immunoassay kit (EIA, Enzo Life Sciences; catalogue no. ADI-901–153), following a previously validated protocol on bonobo samples^47^.

Based on previous studies on humans, chimpanzees, and bonobos, the clearance rate of oxytocin into urine after an event or social interaction is approximately 15 to 60 min^28,47,69,73^. We only included samples which the sampled individual was observed for the entire 90 min prior to sample collection, and we marked whether the sampled individual associated with out-group individuals within the oxytocin clearance window. Our final dataset comprised 601 samples, of which 265 were collected during out-group presence (sampled individual associated with out-group individuals within 15 and 60 min of sample collection) and 336 samples collected during out-group absence (sampled individual only associated with in-group individuals for the entire day). We were conservative in selecting samples for analyses in the context of out-group absence to avoid any anticipatory response prior to intergroup interactions, as shown in chimpanzees^28^. Given that oxytocin activity is linked to other social interactions such as grooming, food sharing, and socio-sexual behaviors^47,48,74^, we also recorded the occurrence of such social interactions within the oxytocin clearance time window. None of the sampled individuals experienced coalitions (given its relatively rare occurrence), food sharing, socio-sexual behaviors, or aggression within 60 min of sample collection. In 81 of the 601 samples (13.5%), the sampled individual engaged in grooming within 60 min of sample collection (55 during out-group absence; 26 during out-group presence).

We also measured specific gravity (SG) of each sample using a digital refractometer (TEC, Ober-Ramstadt, Germany) to correct for the variation in urine concentration across samples. The population mean of SG for bonobos in Kokolopori was 1.0135. We excluded four samples with SG lower than 1.002 because the low SG prevented a reliable correction for urine concentration. All SG corrected urinary oxytocin levels were expressed in pg/ml SG.

### Statistical analyses

#### Individual likelihood to participate in coalitionary aggressions with in-group partners

We investigated whether individual tendency to cooperate with in-group members varied in relation to out-group presence using a generalized linear mixed model (GLMM^75^) with binomial error structure and logit link function^76^. Our previous work examining adult males’ likelihood to cooperate during within- and between-group contexts revealed similar cooperation tendencies in both contexts^53^. Here, we expanded our dataset spanning across five years and included males and females as young as 10 years old because these individuals typically begin to gain non-kin social partners and actively participate in coalitionary aggressions (Cheng, personal observations). At Kokolopori and Wamba, another bonobo research site, males are more involved in intergroup aggression than females^36,41^, while females in Wamba mostly engage in affiliative interactions and cooperate with out-group individuals^38^. This suggests potential sex differences in behavioral strategies during bonobo intergroup encounters. We therefore also explore sex-specific patterns in coalitionary aggression participation (and oxytocin activity). To avoid ambiguity, we excluded 1362 observations that involved unidentified (N = 329) or out-group coalition partners (N = 1033) from the analysis. This resulted in a total of 2544 observations for females (out-group absence: N = 1388; out-group presence: N = 1156) and 847 observations for males (out-group absence: N = 448; out-group presence: N = 399). The response variable in this model was whether a given individual participated in coalitionary aggressions (1) or not (0). The test variables were whether out-group individuals were present (1) or not (0), individual sex, and their interaction. Differences in energetic demands and reproductive tactics may influence individual coalitionary aggression participation. To reliably evaluate the effect of the variables of interest, we included a set of additional control predictors including individual dominance rank (see Supplementary Information for more details), recipient dominance rank, and their interaction, as well as recipient sex. We accounted for the increased opportunity to form in-group coalitions when there were more in-group members present by including the number of in-group individuals present in the party as an offset term.

#### Variation in urinary oxytocin

We investigated whether male and female oxytocin concentration varied in relation to out-group presence using a linear mixed model (LMM^75^) with a Gaussian error structure and identity link. The response variable was the log-transformed oxytocin concentration corrected for SG. To examine female and male oxytocin activity in the context of out-group absence and out-group presence, we included the interaction of the sample context (out-group presence: yes/no) and the sex of the sampled individual as test predictors. We accounted for factors that may influence individual oxytocin concentration, including whether the individual engaged in grooming within the oxytocin clearance window (yes/no), individual dominance rank, in-group party size, group identity (Ekalakala/Kokoalongo), and the amount of urine assayed (200/300/400/500 μl; see Supplementary Information for more details).

## Supplementary Information

Supplementary Information.

## Data Availability

Data used in this study are available on https://figshare.com/s/3e2bc1a703e72a4a1dae.
