# Supplementary material for: Oxytocin activity is not linked to out-group prosociality in wild bonobos
Source: Sci Rep. 2025 Jun 3;15:19408. doi: 10.1038/s41598-025-00209-w (PMC12134060; doi:10.1038/s41598-025-00209-w)
Supplement: Supplementary file 1 — Supplementary Information. [file 41598_2025_209_MOESM1_ESM.docx]

**Oxytocin activity is not linked to out-group prosociality in wild bonobos**

Leveda Cheng, Liran Samuni, Tobias Deschner, and Martin Surbeck

**Supplementary Information**

**Supplementary methods**

Dominance rank assessment

We assessed dominance relationships between two individuals based on the behavioral response upon receiving aggression that represented signs of submission to the aggressor (i.e. fleeing, jumping aside and moving away). We only consider directed dyadic agonistic interactions involving individuals of known identity for rank assessment.

Due to differences in the length of observation period, we used two different methods to calculate dominance ranks for the coalitionary aggression dataset and oxytocin dataset. For the coalitionary aggression dataset, we derived individual dominance ranks using dyadic agonistic interactions between 2016 and 2021 (N = 1983). We calculated a daily dominance rank score for each individual using the Elo-rating method ^1^, which was previously implemented in this population. We extracted individual rank scores, standardized daily in each study group, using extract_elo function in the ‘EloRating’ package ^2^. We set the k factor at 100 and the starting values at 1000. For the oxytocin dataset, we calculated an overall dominance rank score for each individual using the randomized Elo-rating method ^3^, which was previously implemented in this population ^4^. We then extracted the ordinal ranks of individuals from the Elo rank scores and standardized these ordinal ranks separately for each study group. All standardized rank scores ranged from 0 to 1, with 1 being the highest-ranking individual within the group.

Urine sample storage and analysis

In total, we assayed 601 urine samples from 13 males and 20 females. To prevent oxytocin degradation in the samples, we followed the same protocol used by Crockford and colleagues ^5^ and transferred the amount of urine available (500 μl for 74% of the samples; 400 μl for 5% of the samples; 300 μl for 4% of the samples; 200 μl for 17% of the samples) into cryotubes containing 100 μl of 0.1% phosphoric acid using a pipette within 15 mins of sample collection. We stored all samples frozen in liquid nitrogen within 12 h of collection.

All samples were shipped on dry ice to the Endocrinology Lab at the Max Planck Institute for Evolutionary Anthropology and stored at −20 °C until extraction. We extracted urinary oxytocin using a solid-phase extraction with Chromabond HR-X SPE cartridges (1 ml, 30 mg), following a previously validated and published protocol on bonobo samples ^6^. Precisely, we vortexed and centrifuged thawed samples for 5 min (3000 rpm). We diluted thawed urine samples 1:2 using 0.1% trifluoroacetic acid (TFA) in water (TFA-H2O) and loaded onto the cartridge, which was primed with 1 ml methanol and 1 ml HPLC water. We washed the cartridge with 1 ml 10% acetonitrile containing 1% TFA in water and eluded using 1 ml 80% acetonitrile solution. After, we evaporated the extracted samples with air stream at 50 °C before resuspending them in 300 μl 100% ethanol. We then kept the samples at 4 °C for 30 mins and re-evaporated them using the same procedure. The dried samples were reconstituted in 250 μl of the assay buffer supplied in the commercially available enzyme immunoassay kit (EIA, Enzo Life Sciences; catalogue no. ADI-901-153). Samples were added as 100 μl duplicates to the assay, following the instructions of the assay provider. Extracted urine samples with measurements that were outside of the linear range of the oxytocin standard curve were repeated, applying less volume if the original measurement was above the linear range, and more volume when it was below the linear range.

The assay standard curve ranged from 15.62 to 1,000 pg/ml, and assay sensitivity was 15 pg/ml. The inter-assay coefficients of variation (CV) for high and low concentration quality controls (QCs) were 10.8% (QC high: 226.2 pg/ml) and 25.5% (QC low: 52.9 pg/ml), respectively. The intra-assay CV, as calculated by averaging variability across duplicates of all samples measured on a single assay plate, were 12.5% (QC high) and 22.9% (QC low). When the optical density values of sample duplicates differed by more than 10% or when the measurement was outside of the linear range of the assay standard curve, we repeated the measurement. We excluded 190 samples that had insufficient volume for re-measurement or were still outside of the assay linear range after re-measurement.

Statistical model implementation

We fitted the model using the function ‘glmer’ and ‘lmer’ of the package ‘lme4’ in R version 4.4.1 ^7^. We z-transformed all quantitative predictor variables to a mean of zero and standard deviation of one to achieve an easier interpretable model ^8^ and ease model convergence. To test the overall effect of our test predictors and to avoid ‘cryptic multiple testing,’ we conducted a full-null model comparison using a likelihood ratio test, with the null model being identical to the full model but lacking the test predictors (R function ‘anova’ with argument test set to ‘Chisq’ ^9^). For the coalitionary aggression model, we determined the significance of fixed effects by dropping them from the model one at a time ^10^, using the function ‘drop1’. We conducted a post hoc analysis to test the effect of out-group presence and individual sex in relation to each other, using the function ‘glht’ of the package ‘multcomp’ ^11^. For the oxytocin model, we tested the significance of individual fixed effects using the Satterthwaite approximation ^12^, with the function ‘lmer’ of the package ‘lmerTest’ ^13^ and a model fitted with restricted maximum likelihood. We determined confidence intervals of estimates and the fitted models by means of a parametric bootstrap (function ‘bootMer’ of the package ‘lme4’). We examined collinearity using variance inflation factors ^14^ (VIF) with the function ‘vif’ of the package ‘car’ ^15^ and confirmed the absence of collinearity among fixed effects (coalitionary aggression model: maximum VIF = 1.49; oxytocin model: maximum VIF = 1.08). We also ensured that the oxytocin model (LMM with Gaussian error structure) fulfilled the assumptions of normally distributed and homogeneous residuals by visually inspecting a q-q plot ^16^ and the residuals plotted against fitted values ^14^. Finally, we assessed model stability using a function kindly provided by Roger Mundry, which excluded random effect levels one at a time from the data and compared the model estimates derived from these subsets of the data with those derived from the full data set ^17^. This revealed no influential cases in our data set.

Model random effect structure

To account for nonindependence of data points, we incorporated a random effect structure and included individual identity and sample collection day (for the oxytocin model) as random intercept effects. To keep type I error rate at the nominal level of 5% ^10^, we included all theoretically identifiable random slopes in all models (coalitionary aggression model: the presence of out-groups, recipient sex, individual dominance rank, recipient dominance rank, and their interaction within individual identity, the presence of out-groups, individual sex, and their interaction, individual dominance rank, recipient dominance rank, and their interaction within recipient identity, individual dominance rank, recipient dominance rank, and their interaction within dyad, as well as individual sex and individual dominance rank within event identity; oxytocin model: the presence of out-groups, grooming (yes/no), in-group party size, and urine amount within individual identity, as well as individual dominance rank and in-group party size within sample collection day).

**Supplementary references**

1. Neumann, C. *et al.* Assessing dominance hierarchies: validation and advantages of progressive evaluation with Elo-rating. *Anim. Behav.* **82**, 911–921 (2011).

2. Neumann, C. & Kulik, L. EloRating - a brief tutorial.

3. Sánchez-Tójar, A., Schroeder, J. & Farine, D. R. A practical guide for inferring reliable dominance hierarchies and estimating their uncertainty. *J. Anim. Ecol.* **87**, 594–608 (2018).

4. Cheng, L. *et al.* Variation in aggression rates and urinary cortisol levels indicates intergroup competition in wild bonobos. *Horm. Behav.* **128**, 104914 (2021).

5. Crockford, C. *et al.* Urinary oxytocin and social bonding in related and unrelated wild chimpanzees. *Proc. R. Soc. B Biol. Sci.* **280**, (2013).

6. Moscovice, L. R. *et al.* The cooperative sex: Sexual interactions among female bonobos are linked to increases in oxytocin, proximity and coalitions. *Horm. Behav.* **116**, 104581 (2019).

7. Bates, D., Mächler, M., Bolker, B. & Walker, S. Fitting linear mixed-effects models using *lme4*. *J. Stat. Softw.* **67**, (2015).

8. Schielzeth, H. Simple means to improve the interpretability of regression coefficients. *Methods Ecol. Evol.* **1**, 103–113 (2010).

9. Forstmeier, W. & Schielzeth, H. Cryptic multiple hypotheses testing in linear models: Overestimated effect sizes and the winner’s curse. *Behav. Ecol. Sociobiol.* **65**, 47–55 (2011).

10. Barr, D. J., Levy, R., Scheepers, C. & Tily, H. J. Random effects structure for confirmatory hypothesis testing: Keep it maximal. *J. Mem. Lang.* **68**, 255–278 (2013).

11. Bretz, F., Hothorn, T. & Westfall, P. *Multiple Comparisons Using R*. (CRC Press, Boca Raton, FL, 2016). doi:10.1201/9781420010909.

12. Luke, S. G. Evaluating significance in linear mixed-effects models in R. *Behav. Res. Methods* **49**, 1494–1502 (2017).

13. Kuznetsova, A., Brockhoff, P. B. & Christensen, R. H. B. lmerTest package: Tests in linear mixed effects models. *J. Stat. Softw.* **82**, 1–26 (2017).

14. Quinn, G. P. & Keough, M. J. *Experimental Design and Data Analysis for Biologists*. (Cambridge University Press, Cambridge, 2002).

15. Fox, J. & Weisberg, S. *An R Companion to Applied Regression. (2 Ed.)*. (Sage, Thousand Oaks, 2011).

16. Field, A. *Discovering Statistics Using SPSS, 2nd Ed*. (Sage Publications, Inc, Thousand Oaks, CA, US, 2005).

17. Nieuwenhuis, R., te Grotenhuis, M. & Pelzer, B. Influence. ME: Tools for detecting influential data in mixed effects models. *R-J.* **4**, 38–47 (2012).
